# Psoriatic Synovitis: Singularity and Potential Clinical Implications

**DOI:** 10.3389/fmed.2019.00014

**Published:** 2019-02-11

**Authors:** Raquel Celis, Andrea Cuervo, Julio Ramírez, Juan D. Cañete

**Affiliations:** Arthritis Unit, Rheumatology Department of Hospital Clinic and IDIBAPS, Barcelona, Spain

**Keywords:** psoriatic arthritis, rheumatoid arthritis, synovitis, immunohistochemistry, macrophage-polarization, microarrays, mass spectrometry image

## Abstract

Psoriatic arthritis (PsA) is an immuno-inflammatory disease with a heterogeneous clinical presentation as affects musculoskeletal tissues (arthritis, enthesitis, spondylitis), skin (psoriasis) and, less frequently, eye (uveitis) and bowel (inflammatory bowel disease). It has been suggested that distinct affected tissues could exhibit different immune-inflammatory pathways so complicating the understanding of the physiopathology of psoriatic disease as well as its treatment. Despite of the key pathogenic and clinical relevance that enthesitis has in PsA, peripheral arthritis is more easily perceived. At the macroscopic level, PsA synovitis has predominantly tortuous, bushy vessels, whereas rheumatoid arthritis (RA) is characterized by mainly straight, branching vessels so reflecting prominent neo-angiogenesis in PsA. Synovial biopsies have demonstrated a similar cellular and molecular picture in PsA and RA, although some differences have been reported at the group level, as higher density of vessels, CD163+ macrophages, neutrophils and mast cells in PsA. In fact, synovial IL-17+ mast cells are significantly increased in PsA and produce more IL-17A compared with RA, and a proof of concept study supports its relevant role in the synovitis of SpA, included PsA. As firstly reported in RA, synovial lymphoid neogenesis is found also in the same proportion of PsA as in RA patients, despite the lack of autoantibodies in PsA. These lymphoid structures are associated with activation of the IL-23/Th17 pathway in RA and seemly in PsA, which could be useful to stratify RA patients. Immunohistochemical and transcriptomic methodologies have still not found synovial biomarkers useful to distinguish psoriatic from rheumatoid synovitis at the patient level. However, modern methodologies, as MALDI-Mass Spectrometry Imaging, applied to the study of synovial tissue have revealed metabolic and lipid signatures which could support clinical decision-making in the diagnosis of PsA and RA and to go further toward the personalized medicine.

## Introduction

Psoriatic arthritis (PsA) is an immune-mediated inflammatory disease with a wide range of clinical manifestations: synovitis, enthesitis, spondylitis, dactylitis, skin, and nail psoriasis. More rarely, it involves the eye (uveitis) and the bowel (Crohn's disease). PsA is included in the spondyloarthritis (SpA) concept, which encompasses a group of diseases sharing immunogenetic, pathophysiological, clinical, and radiological features, which differ from rheumatoid arthritis (RA) (1). McGonagle et al. hypothesized that the primary lesion of SpA is enthesitis, that enthesopathy may be the common link between all forms of SpA, and that enthesitis in SpA synovial joints is frequent (2, 3). The close anatomical relationship between the enthesis, prone to mechanical stress, and the vascular synovium, in contact with a variety of immune mediators, may provide the pathogenic basis for joint inflammation in SpA, including PsA. The functional unit formed by enthesis and adjacent synovium was termed as synovial enthesis complex (SEC). The SEC represents a conceptual framework, which may explain the tissue specificity and highlights the role of mechanical stress in SpA, while at the same time providing a unifying pathophysiological concept for PsA based on the idea that specific tissues may be particularly sensitive to mechanical triggers (4). Paramarta et al. challenged the hypothesis of enthesitis being the primary lesion in SpA leading to a secondary synovitis over time, although the authors recognized some limitations in their study (5). Also, the study of enthesitis pathophysiology is limited by the difficulty to obtaining biopsies from the enthesis due to potential adverse effects.

Arthritis is more easily perceived that enthesitis, as clinical trials and registries of patients with PsA have showed. Peripheral arthritis is a key target of the pathogenic process which may lead to joint destruction and associated impaired function and quality of life (6). Therefore, psoriatic synovitis has been widely studied, generally as part of other peripheral SpA and has been compared with RA, the most prevalent peripheral arthritis (7).

The synovial membrane (synovium) borders the joint cavity and attach to the bone-cartilage interface. A healthy synovium consists of a thin layer (lining) 1-2 cells thick containing synovial fibroblasts and macrophages. Below this layer is the sublining, which is composed of loose connective tissue with blood vessels, lymphoid vessels, fibroblasts, nerve fibers, and few leucocytes. The inflamed synovium (synovitis) has three histological characteristics: lining hyperplasia (proliferation of synovial fibroblasts and accumulation of macrophages); neoangiogenesis (blood vessel proliferation in the sublining), and huge infiltration of the sublining by inflammatory cells, including lymphocytes, macrophages, dendritic cells and mast cells, which produce proinflammatory cytokines, growth factors and metalloproteases contributing to persistent synovitis and joint destruction (1, 7).

The study of synovitis in PsA, RA and other chronic arthritis is being driven by mini-arthroscopy and ultrasound-guided biopsies, which are safe and well-tolerated techniques and allow synovial tissue samples to be obtained from large and small joints at any stage of activity of disease: early, established, active or remission, as well as before and after therapeutic interventions. Taken together, easier extraction of synovial tissue together with the application of powerful new methodologies (trancriptomics, single-cell RNA, proteomics, metabolomics, new inmmunohistologic markers, mass spectrometry image analysis) will accelerate the study of synovitis to better understand their diagnostic and prognostic implications (8). Most studies on synovitis have focused on RA, while others comparing RA and SpA, included PsA; however few studies have focused specifically on PsA. We review PsA synovitis from the macroscopic (arthroscopy) and microscopic perspective, highlighting the cellular and molecular characteristics of each of the histological alterations of synovitis mentioned above as compared with RA. Table 1 displays some key clinical and pathogenic differences between PsA and RA.

**Table 1:** Differences between PsA and RA.

**Features**	**Psoriatic arthritis**	**Rheumatoid arthritis**
Clinical	Asymmetrical arthritis lower limbs DIP joints, enthesitis, dactylitis, Axial arthritis	Symmetrical MCP and wrist joints
Genetics	HLA-B38, -B39, and HLA-B27 HLA-cw6 IL-23/IL-17 pathway-related genes	HLA-DRB1 PTPN22
Pathogenesis		
Autoantibodies	No	ACPA and RF
Synovial immunopathology	Innate immune cells (IL-17A+ Mast cells, Neutrophils)	Adaptive immune cells (B and T-cells)
Synovial neoangiogenesis	Intense	Moderate
Vessels morphology^*^	Bushy, tortuous vessels	Straight, branching vessels
Radiology	Bone erosion and neoformation	Erosion
Therapeutic targets	TNFi Anti-IL-23/IL-12 Anti-IL-17A Anti-IL-23	TNFi Anti-B cells (anti-CD20) Anti-T cells (CTL4-Ig) Anti-IL-6

MCP, Metacarpophalangeal; DIP, distal interphalangeal; ACPA, Anti-Citrullinated Peptide Antibody; RF, Rheumatoid Factor; IL, Interleukin; TNFi, Tumor Necrosis Factor inhibitors;

* *Macroscopy vessels morphology as seen by arthroscopy*.

## Synovial Tissue Features in PsA Synovitis

The morphologic and cellular heterogeneity of synovitis requires review of the macroscopic features, which appear to differ between PsA and RA, and subsequent description of the cellular features according to the key changes that occur in inflammation: lining hyperplasia, neo-angiogenesis and leukocyte infiltration.

### Macroscopic Features of PsA Synovitis

**Figure 1:**
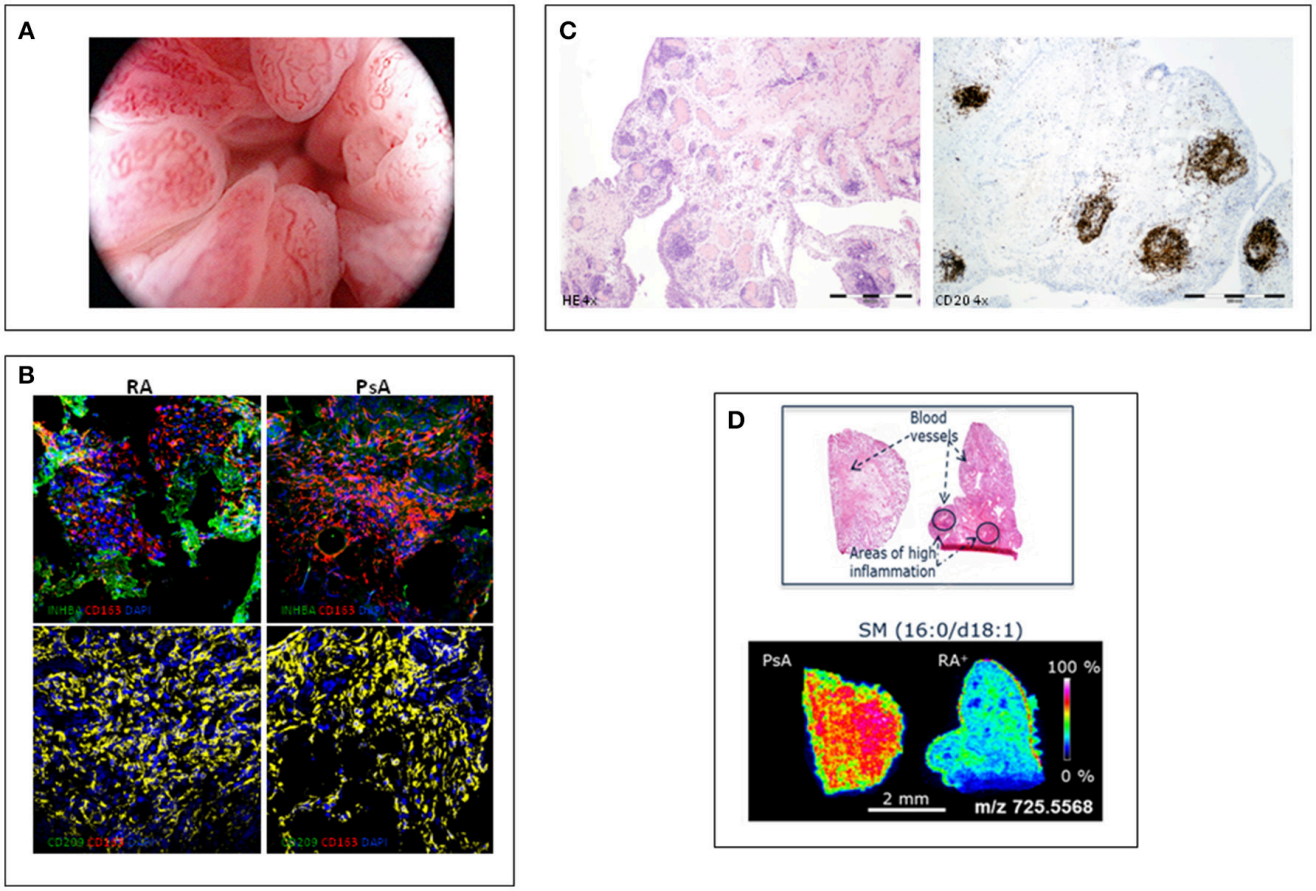
Different features of psoriatic synovitis are represented. **(A)** Arthroscopic view of psoriatic synovitis with erithematous villae plenty of dilated, tortuous vessels. **(B)** Immunofluoresence analysis of the expression of macrophage-polarization markers in synovial tissue CD163+ macrophages from RA and PsA patients, as determined by confocal microscopy using anti-INHBA (Activin A) –a GM-CSF induced gene- and CD209 –a M-CSF induced gene- specific antibodies; nuclei were counterstained with DAPI (Courtesy of A Puig-Kröger, PhD, Madrid, Spain). **(C)** Staining of inflammed synovium from a patient with PsA; left: H-E staining (4x) showing a general view of synovial membrane standing out abundant vessels surrounded by folicular aggregates; right: CD20 staining (4x) highlighting the B-cell folicles in PsA synovitis. **(D)** Mass Spectometry Image analysis showing spatial mapping positive-lipid ion in synovium sections of PsA and RA. Scale bar shows normalized intensities (Courtesy of Prof. F Blanco, A Coruña, Spain).

Using rheumatologic arthroscopy, Reece et al. (9) found significant differences in the pattern of new blood vessel between psoriatic and rheumatoid synovitis. PsA synovitis is characterized by erythematous villae with dilated, bushy and tortuous vessels (Figure 1A) whereas RA synovitis predominantly shows straight, branched vessels. This distinct pattern probably reflects a distorted proliferation of neovessels (neo-angiogenesis) due to increased expression of pro-angiogenic mediators, such as VEGF and Angiopoietin-2 (Ang-2), in PsA (10). Other studies confirmed these findings in PsA and peripheral SpA, with some differences in the frequency of the straight and branched pattern of RA (11–13). Despite its high sensitivity and specificity, the bushy and tortuous pattern is not diagnostic of PsA, although it may be a useful guide in the diagnostic work-up of undifferentiated arthritis (12).

### Cellular Features of PsA Synovitis

#### Hyperplasia of the Synovial Lining

Fibroblast-like synoviocytes (FLS) and macrophages are the cellular components of the lining. Inflammation induces activation, proliferation and/or diminished FLS apoptosis, whereas macrophages increase due to infiltration from the peripheral blood. Studies of FLS in PsA are scarce compared with RA, where it has been shown that FLS undergo epigenetic changes, becoming persistently activated and forming the invasive front of synovial tissue in the joint cartilage (pannus) (14). A study of the effects of Janus kinase inhibitor tofacitinib on synovial fibroblast function suggested that PsA fibroblasts are activated similarly to RA fibroblasts (15). In RA, FLS change phenotypically and functionally at different anatomical sites and contribute to the identity of individual tissue, and they are capable of actively participating and orchestrating inflammation and immunity (14). A single-cell RNA sequencing and immunohistochemistry based study has described three functionally distinct subsets of FLS in RA: lining, immunoregulatory, and pathogenic fibroblasts populations. Pathogenic fibroblasts are located in the sublining around the vessels (CD34-CD90+) and they are the only FLS subset significantly increased in RA compared with osteoarthritis (16). Although several studies have reported increased lining hyperplasia in RA compared with PsA, others have found no differences (7). Using the Hsp47 antibody, a new specific marker of lining and sublining FLS (17), we have found a significant increase of sublining FLS, but no lining FLS, in RA compared with PsA, without between-group differences in systemic inflammation markers (CRP) (18). Lining CD68+ macrophages are functionally heterogeneous and include proinflammatory and tissue resident macrophages, a population not well-defined by lack of markers, but there are no differences in their cellular density between PsA and RA synovitis (19).

#### Neoangiogenesis

In line with the macroscopic hypervascularization that characterizes PsA synovitis, several studies have found an increase of vessels density in PsA compared with RA. Furthermore, different pro-angiogenic factors are expressed in the two diseases, with increased Ang-2 in PsA and Ang-1 in RA (20–22). Successful treatment with anti-TNF therapy in PsA synovitis reduces expression of VEGF and its receptors VGFR1 and VGFR2, but not Ang-2 expression, leading to regression of neovessels, probably by inducing endothelial cell apoptosis (23).

A recent study comparing CD31+ synovial vessels between PsA (*n* = 38) and RA (*n* = 40) patients found no significant differences between the two diseases (18).

#### Synovial Leukocyte Infiltrate

A vast influx of inflammatory cells of the innate and adaptive immune system populates the inflamed synovial membrane, with the most being macrophages, neutrophils, mast cells, and T and B-lymphocytes. All these cells are activated and produce multiple pro-inflammatory and pro-angiogenic cytokines, chemokines, growth factors, metalloproteases, and other mediators, which contribute to the persistence of synovitis and joint destruction. Global cell infiltration in PsA and RA synovitis in the histologic analysis is similar, although characterization by immunohistochemistry of the infiltrating cells could encounter differences, as synovial infiltration by mast cells, CD15+ neutrophils and CD163+ macrophages is increased in SpA, included PsA, compared with in RA (7).

#### Synovial Macrophages

CD68+ macrophages accumulate in the synovium of RA and PsA joints, where they exhibit destructive and remodeling potential and contribute considerably to joint inflammation and joint destruction (24, 25). In RA and in SpA, including PsA, macrophage density correlates with disease activity (19). Sublining CD68+ macrophages density has been shown to be similar in PsA and RA synovitis (18, 26). A small study comparing RA and PsA synovitis found that synovial p53 expression and CD68+ macrophages density was associated with erosive disease only in RA suggesting that CD68+ macrophages differ in the destructive potential between RA and PsA (27).

Few studies have analyzed macrophage subsets in chronic arthritis, but have shown differences, probably due to the markers used. CD163-positivity has been proposed as a biomarker of anti-inflammatory macrophages and CD163+ macrophages were found overexpressed in SpA synovitis, whereas RA was characterized by overexpression of pro-inflammatory macrophage markers (19). A study using surface markers (CD14, CD163, CD68, CD32, CD64, CD200R, CD80) on synovial tissue macrophages from RA and SpA patients found that macrophages had a mixed M1-proinflammatory/M2-anti-inflammatory phenotype, with M1 predominance in RA and IL-10-expressing macrophages in SpA (28).

The characterization of *ex-vivo* CD14+ macrophages isolated from the synovial fluid of patients with active RA indicates that they exhibit a transcriptomic and protein profile that is compatible with a GM-CSF-skewed macrophage polarization (29). The proteins encoded by several of the GM-CSF-associated gene markers have also been detected in macrophages from active RA synovial tissue, including activin A, MMP12 and CCR2 (29). We analyzed the expression of markers of GM-CSF derived macrophages (INHBA, MMP12, and TNFα) and M-CSF derived macrophages (CD209) on CD163+ macrophages, and found a similar expression of GM-CSF- and M-CSF-associated markers in synovial tissue of RA and PsA patients (30) (Figure 1B). These results support the presence of similar GM-CSF and M-CSF skewed macrophages in RA and PsA synovitis.

#### Synovial Mast Cells

Mast cells have been reported to have a potential sentinel function as innate protective cells which is supported by their strategic location in skin, gut, and airways, and their expression of specific danger signal receptors such as TLR2 and TLR4. Mast cells also have the ability to synthesize and, in addition, release preformed mediators including cytokines, proteases, and anti-microbial defensins (31). Mast cells play a previously- unappreciated role in synovial inflammation in SpA, included PsA, as it has been shown that they are significantly more abundant in PsA than in RA synovitis and, importantly, they are also the main cellular source of IL-17 A in PsA synovial tissue. These findings are independent of the disease stage and anti-TNF therapy (32). However, the absence of IL-17A mRNA in mast cells has also been demonstrated and a novel mechanism whereby mast cells capture and store exogenous IL-17A in specialized intra-cellular vesicles through receptor-mediated endocytosis, releasing bioactive IL-17 A after mast cell stimulation, has been discovered (33).

New findings reporting IL-17A-loaded mast cells in the normal skin and gut, in SpA synovial tissue before and after anti-IL-17 A antibody secukinumab, and in the inflamed gut, support the concept of mast cells as sentinel cells, as IL-17A-positive mast cells are readily available in non-inflamed tissues, and the IL-17A content decreased during inflammation in the gut lamina propria and increased upon anti-inflammatory treatment of SpA synovitis (31). Therefore, the presence of IL-17A-positive mast cells across different SpA target tissues and the inverse correlation between their IL-17A-content and inflammation indicate that the IL-17A content in mast cells can be regulated (31). Understanding how IL-17A can be controlled locally during tissue inflammation may result in novel therapeutic strategies to target IL-17A, a key cytokine in PsA (31).

In RA synovitis, high synovial mast cell counts are associated with local and systemic inflammation, autoantibody positivity and high disease activity. They are located at the outer border of lymphoid aggregates. Furthermore, mast cells promote the activation and differentiation of naïve B cells and induce ACPA production, mainly via contact-dependent interactions (34). Although synovial mast cells are also the main IL-17A positive cells in RA synovitis, its role remains to be studied (35).

#### Synovial Neutrophils

Polymorphonuclear cells have been reported to be increased in synovial tissue of axial and peripheral SpA, including PsA synovitis, compared with RA, and correlated with disease activity. Their reduction after treatment was associated with a good therapeutic response, leading to them being defined as a biomarker of response for SpA (36, 37). In fact, after mast cells, neutrophils (CD15+ cells) are the most frequent IL-17 A+ cells in SpA and PsA (32). Neutrophils are scarce in RA synovitis, but a recent study comparing sinovial CD15+ cells (neutrophils) in PsA and RA synovitis found no significant differences between the two diseases (18).

#### Lymphocytes and Ectopic Lymphoid Neogenesis

Although PsA seems to have a partial autoinflammatory pathophysiology whereas RA has a strong autoimmunity component (38), in general sinovial T and B-lymphocytes, and plasma cells have been found to be similar in PsA and RA synovitis (7). However, beyond the number and type of infiltrating leukocytes, their spatial organization in the sinovial microarchitecture may be of pathophysiological relevance (7). Ectopic lymphoid neogenesis (ELN) is characterized by lymphocyte aggregates (Figure 1C) with prototypical features that recapitulate those of germinal centers, such as the presence of high endotelial venules and folicular dendritic cells (39). As ELN resembles secondary lymphoid tissues, it has been proposed that sinovial ELN may play a role in mounting immune responses, and specifically the autoimmune response observed in RA (40). However, sinovial ELN is similarly found in PsA and in RA, and there is no association with the presence of RA-specific autoantibodies (41–43). However, synovial ELN in PsA and RA have been associated with a different cytokine profile characterized by specific expression of the IL-23/Th17 cytokines axis (44, 45). These findings suggest that an important subgroup of RA patients express high IL-23/IL-17 cytokines, introducing the potential of stratification of patients by ELN in exploratory clinical trial for anti-IL23 or anti-IL-17 antibodies.

### Microarray Analysis of Synovial Tissue in PsA

Comparison of synovial biopsies of patients with RA and SpA, including PsA, to analyse synovial molecular and cellular processes by pan-genomic microarray, has revealed a myogene signature specific for SpA, which was independent of disease duration, treatment and SpA subtype (non-psoriatic vs. psoriatic). These findings were confirmed by qPCR and immunohistochemistry analysis, and the synovial cells expressing myogenes were identified as vimentin-positive, prolil4-hydroxilase-positive, CD90+,CD146+ mesenchimal cells in the lining and sublining layers. This specific myogene signature did not change after anti-TNF therapy (46).

A study of gene array in paired skin and synovial biopsy samples from 12 patients with both PsA and psoriasis, confirmed by PCR and immunohistochemistry, showed that gene expression patterns in psoriatic skin and synovium differed, with a stronger IL-17 signature in skin than synovium, while TNF was higher in synovium (47). These transcriptomic analysis reveal new molecular pathways that open new avenues in the knowledge of the differential pathogenesis of synovitis in PsA and RA as well as between different tissues involved in PsA.

#### Mass Spectrometry Imaging Analysis

A pionner study used Mass Spectrometry Imaging (MSI) to identify lipid and metabolic profiles in the synovial tissue of 25 patients with PsA, 21 with RA (16 seropositive and 5 seronegative) and 10 with undifferentiated arthritis. Tissue sections were deposited on conductive slides and coated with different matrices for lipid and metabolite extraction. MALDI images were acquired on a rapifleX MALDI Tissuetyper time-of-flight instrument. Multivariate data analysis was used to search for the lipids and metabolites with the highest between-group differences.

MALDI-MSI revealed differentiated lipid and metabolic profiles in all the groups studied. Discriminant analysis of the lipid data acquired in positive ion mode displayed a good separation of patients with PsA and RA, especially seropositive RA (Figure 1D). PsA synovium was characterized by a higher content of phospholipids compared to seronegative and seropositive RA. However, sugar metabolites displayed a stronger intensity in RA than in PsA synovium. Metabolic and lipid signatures reported with this new methodology could support clinical decision-making in the diagnosis of RA and PsA (48).

## Conclusions

Globally, PsA synovitis has more similarities than differences when compared with RA at the histologic and immunohistochemical level. However, there is some singularities in PsA that merit more in-depth research: the role of IL-17-positive mast cells in PsA inflammation and in IL-17 A regulation; the role of ectopic lymphoid neogenesis in PsA, and to know if there is distinct functional subsets of synovial FLS in PsA as in RA. New research tools as pan-genomic microarrays and metabolomics/proteomics associated to mass spectrometry image analysis are full of promise to reveal new cellular and molecular features specific to PsA synovitis which improve our diagnostic and prognostic potential.

## Author Contributions

JC revised the references and wrote the first draft of the manuscript. RC, AC, and JR revised the manuscript and collaborate in the discussion and writing to the last version. All the co-author revised and approved the manuscript.

### Conflict of Interest Statement

The authors declare that the research was conducted in the absence of any commercial or financial relationships that could be construed as a potential conflict of interest.
